# Supplementary material for: In Vivo Lentiviral Gene Delivery of HLA-DR and Vaccination of Humanized Mice for Improving the Human T and B Cell Immune Reconstitution
Source: Biomedicines. 2021 Aug 5;9(8):961. doi: 10.3390/biomedicines9080961 (PMC8393476; doi:10.3390/biomedicines9080961)
Supplement: Supplementary file 1 [file biomedicines-09-00961-s001.zip › 20210727 Supp. tab. Kumar, Koenig et al.pdf]

Supplementary Tables

Table S1: Antibodies used in the studies.

Application	Antigen/Clone	Fluorochrome	Company	Catalogue #	Dilution
**HLA-DR expression *in vitro***	HLA-DR/L243 (data shown)	APC	Biolegend	307609	1:20
	HLA-DR/L203 (data not shown)	PE	R&D Systems	FAB4869P-100	1:20
	HLA-DR4 (data not shown)	Biotin	One Lambda	BIH0453B	1:10
**gB expression *in vitro***	gB/p27-287	-	Kindly provided by Michael Mach, Erlangen, Germany	-	1:5-1:20
	Anti-mouse IgG	AF488	Biolegend	405319	1:250
**Panel 1 for humanized mice (Longitudinal analyses of PBL)**	CD45/HI30	Pacific Blue	Biolegend	304022	1:800
	CD3/UGHT1	BV510	Biolegend	300448	1:100
	CD4/OKT4	PerCP	Biolegend	317432	1:400
	CD8a/HIT8a	PE-Cy7	Biolegend	300914	1:200
	CD19/HIB19	AF700	Biolegend	302225	1:800
**Panel 2 for humanized mice (T cell phenotypes in spleen)**	CD45/HI30	Pacific Blue	Biolegend	304022	1:800
	CD3/UGHT1	BV510	Biolegend	300448	1:100
	CD4/OKT4	PerCP	Biolegend	317432	1:400
	CD8a/HIT8a	PE-Cy7	Biolegend	300914	1:200
	CD45RA/ALB11	FITC	Beckman Coulter	A07786	1:100
	CD62L/DREG56	PE-Cy5	Biolegend	304808	1:200
**Panel 3 for humanized mice (B cell development in spleen)**	CD45/HI30	AF700	Biolegend	304024	1:100
	CD19/HIB19	PerCP-Cy5.5	Biolegend	302230	1:100
	IgM/MHM-88	Pacific Blue	Biolegend	314514	1:100
	IgG/G18-145	PE-Cy7	BD	561298	1:50
	IgA/IS11-8E10	PE	Miltenyi	130-093-128	1:100
**gB ELISA**	anti-human IgG	HRP	Bio-Rad	5172-2504	1:5000

Table S2: Descriptive statistics for figure 5C comparing the bioluminescence signal between the cohorts CTR, 1 μg and 3 μg. Statistical analyses were performed using the t-test (unpaired).

Group	Week Post-HCT	CTR		1 μg		3 μg		P value	
		Mean	SD	Mean	SD	Mean	SD	CTR x 3 μg	1 μg x 3 μg
NRG	2	31700.0	7280.7	177433.3	205271.2	340000.0	141714.5	***0.0197***	0.3221
	7	42733.3	11643.1	296666.6	183063.7	233133.3	203265.4	0.1806	0.708
	12	39893.3	5297.4	349633.3	225067.1	238433.3	87382.0	***0.0171***	0.4697
	15	32833.3	7804.0	73800.0	26130.6	197966.6	147341.1	0.1246	0.224
huNRG	2	24275.0	4299.9	317833.3	413491.0	357575.0	266349.2	***0.0464***	0.882
	7	42750.0	12218.1	230933.3	226091.1	321225.0	243084.0	0.0621	0.6383
	12	42222.5	9714.33.0	117850.0	54194.2	234050.0	45595.9	***0.0002***	***0.0271***
	15	66925.0	6932447.0	128433.3	105039.7	211750.0	79172.2	***0.0332***	0.2816

Table S3: Descriptive statistics for figures 6B and 6C comparing the frequencies of $CD4^+$ and $CD8^+$ T cells in spleen between the cohorts CTR, 1 μg and 3 μg. Statistical analyses were performed using the t-test (unpaired).

Marker	CTR (n=4)		1 µg (n=3)		3 µg (n=4)		P value
	Mean	SD	Mean	SD	Mean	SD	CTR x 3 µg
% CD4	1.23	0.6	1.82	0.43	1.63	0.75	0.4373
% CD8	1.96	2.03	2.17	1.44	2.0	1.19	0.9756

Table S4: Descriptive statistics for figure 7C comparing the bioluminescence signal between the cohorts DR4 and DR4/VAC. Statistical analyses were performed using the Welch t-test applied to log data (unpaired).

Week post-HCT	DR4 (n=11)		DR4/VAC (n=9)		P value
	Mean	SD	Mean	SD	DR4 x DR4/VAC
2	302000	201000	346000	218000	0.6135
7	131000	52000	132000	57000	0.9837
12	63700	23500	72200	21500	0.3419
20	68500	38200	54700	32300	0.3353

Table S5: Descriptive statistics for figures 8B and 8C comparing the frequencies of CD4$^+$ and CD8$^+$ T cells in peripheral blood between the cohorts CTR, VAC, DR4 and DR4/VAC. Statistical analyses were performed using the Welch t-test (unpaired).

Marker	Week post-HCT	CTR (n=6)		VAC (n=7)		DR4 (n=11)		DR4/VAC (n=9)		P value
		Mean	SD	Mean	SD	Mean	SD	Mean	SD	CTR x DR4/VAC
% CD4	10	0.71	0.92	1.10	0.68	1.01	0.79	3.03	6.17	0.2993
	15	8.04	4.96	4.86	2.90	3.95	2.80	9.63	9.20	0.6734
	20-22	21.57	16.48	18.63	17.26	16.34	15.65	19.74	19.91	0.8496
% CD8	10	0.76	0.74	1.01	0.69	1.24	1.41	3.12	5.03	0.2011
	15	6.35	4.83	6.19	3.01	5.30	3.06	7.77	11.56	0.7486
	20-22	9.91	6.67	12.52	8.77	13.75	11.69	11.30	13.71	0.7974

Table S6: Descriptive statistics for figures 9A, 9B, 9C and 9D comparing the total number of CD4$^+$ and CD8$^+$ T cell subsets in spleen between the cohorts CTR, VAC, DR4 and DR4/VAC. Statistical analyses were performed using the Welch t-test applied to log data (unpaired).

Marker	CTR (n=6)		VAC (n=7)		DR4 (n=11)		DR4/VAC (n=9)		P value
	Mean	SD	Mean	SD	Mean	SD	Mean	SD	CTR x DR4/VAC
#CD4/EM	865718	1640415	283477	324709	279185	403883	614735	875404	0.9290
#CD4/TE	23760	18884	46084	43928	34430	29600	57049	45466	**0.0677**
#CD8/EM	225711	433948	155378	243208	86094	83721	503136	992830	0.4276
#CD8/TE	77313	83077	93603	115551	128694	277602	129995	145667	0.3281

Table S7: Descriptive statistics for figures 10A, 10B and 10C comparing the total number of CD19$^+$/IgG$^+$, CD19$^+$/IgA$^+$ and CD19$^+$/IgM$^+$ cells in spleen between the cohorts CTR, VAC, DR4 and DR4/VAC. Statistical analyses were performed using the Welch t-test applied to log data with Laplace correction (+1) (unpaired).

	CTR (n=6)		VAC (n=7)		DR4 (n=11)		DR4/VAC (n=9)		P value
	Mean	SD	Mean	SD	Mean	SD	Mean	SD	CTR x DR4/VAC
**#CD19/IgG in SPL**	5735	4473	20949	21068	16593	28896	27025	22004	***0.01252***
**#CD19/IgA in SPL**	13716	13105	50843	57272	36768	53438	57327	45567	***0.00123***
**#CD19/IgM in SPL**	2051425	1082920	3488884	2841114	2369652	2283161	3209464	2333836	0.5433

Table S8: Descriptive statistics for figures 11A and 11B comparing the total number of cells in LNs and detectable IgG against gB in plasma between the cohorts CTR, VAC, DR4 and DR4/VAC. Statistical analyses were performed using the Welch t-test applied to log data.

		CTR (n=6)		VAC (n=7)		DR4 (n=11)		DR4/VAC (n=9)		P value	
		Mean	SD	Mean	SD	Mean	SD	Mean	SD	CTR x DR4/VAC	DR4 x VAC
**#Cells in LNs**		351667	348162	431429	481402	735455	712690	724444	517183	0.1375	-
**O.D.**	**1/5 Dilution**	0.3	0.3	0.78	0.63	0.27	0.18	0.26	0.10	-	***0.02998***
	**1/25 Dilution**	0.16	0.13	0.44	0.38	0.14	0.05	0.16	0.05	-	***0.02708***
