# Supplementary material for: In Vivo Lentiviral Gene Delivery of HLA-DR and Vaccination of Humanized Mice for Improving the Human T and B Cell Immune Reconstitution
Source: Biomedicines. 2021 Aug 5;9(8):961. doi: 10.3390/biomedicines9080961 (PMC8393476; doi:10.3390/biomedicines9080961)
Supplement: Supplementary file 1 [file biomedicines-09-00961-s001.zip › 20210803 Supp. fig. Kumar, Koenig et al.pdf]

biomedicines

MDPI

Supplementary figures

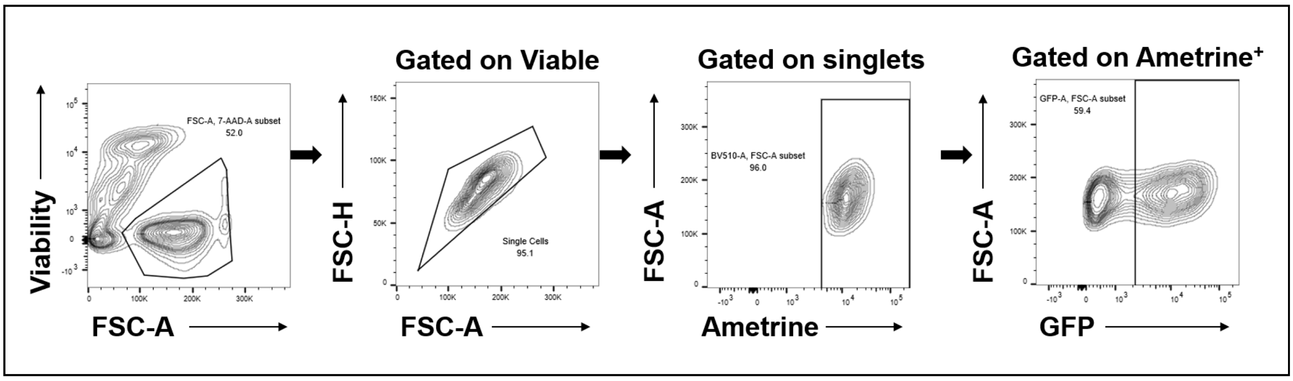

Figure S1. FACS gating strategy for quantifying frequencies of GFP+ 58-T cells. Representative example from the co-culture of 58-T cells (non-adherent) with 3T3 cells (adherent) stimulated with a cognate peptide (GAD).

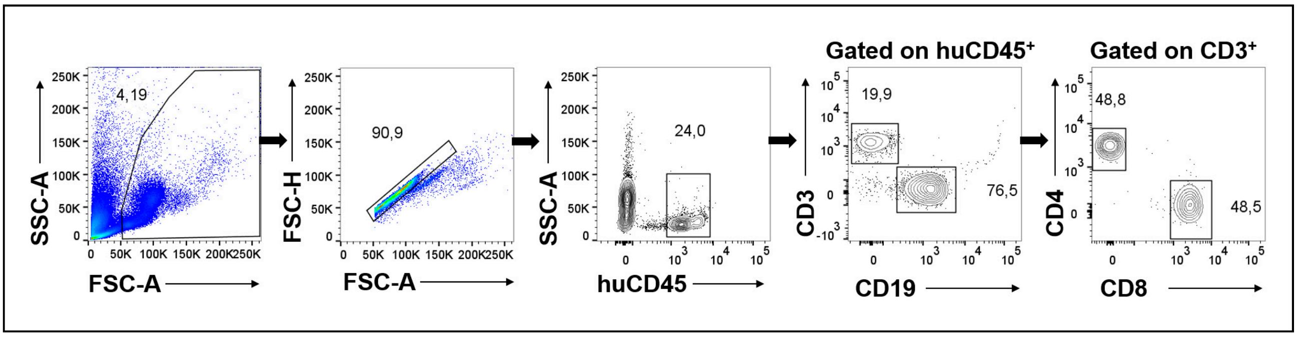

Figure S2. FACS gating strategy for immune-reconstitution analyses of T and B cells in peripheral blood. Representative example of huNRG blood analyses 20 weeks after HCT for an DR4/VAC mouse.

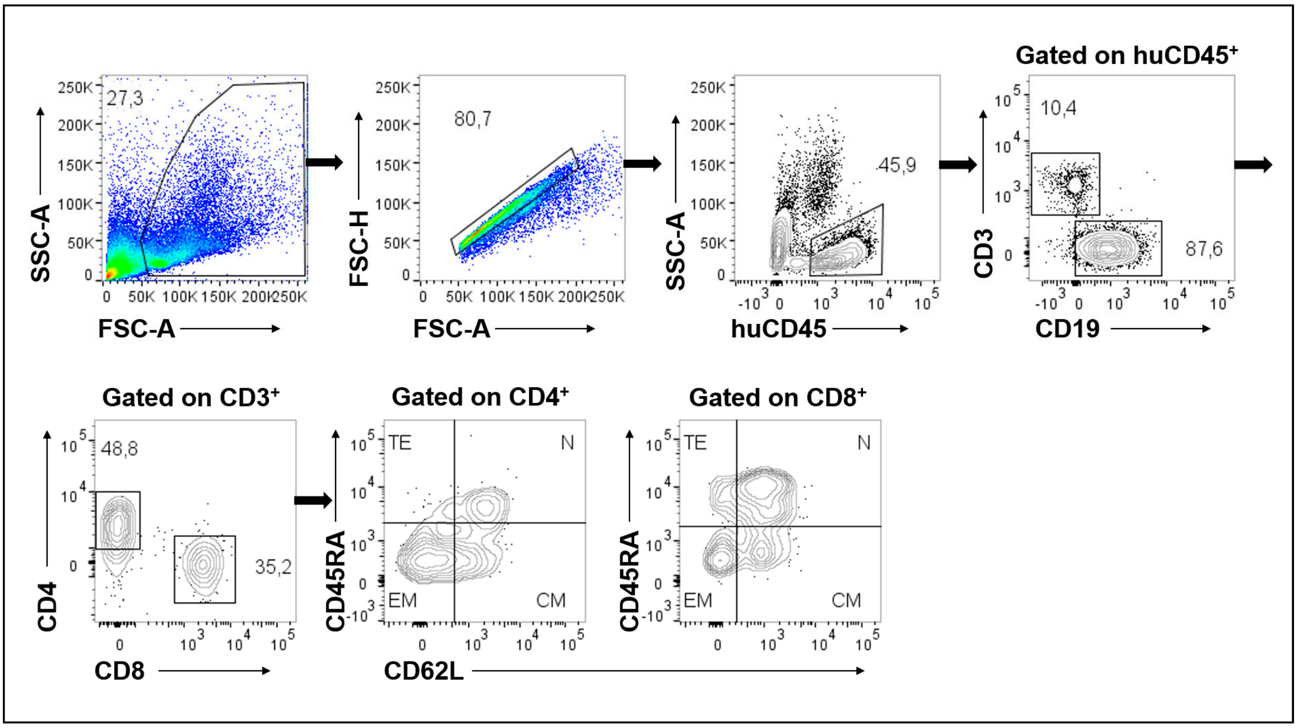

Figure S3. FACS gating strategy for analyses of T cell phenotype in spleen. Representative example of huNRG spleen analyses 20 weeks after HCT for an DR4/VAC mouse. Naïve (N), central memory (CM), effector memory (EM), terminal effector (TE)

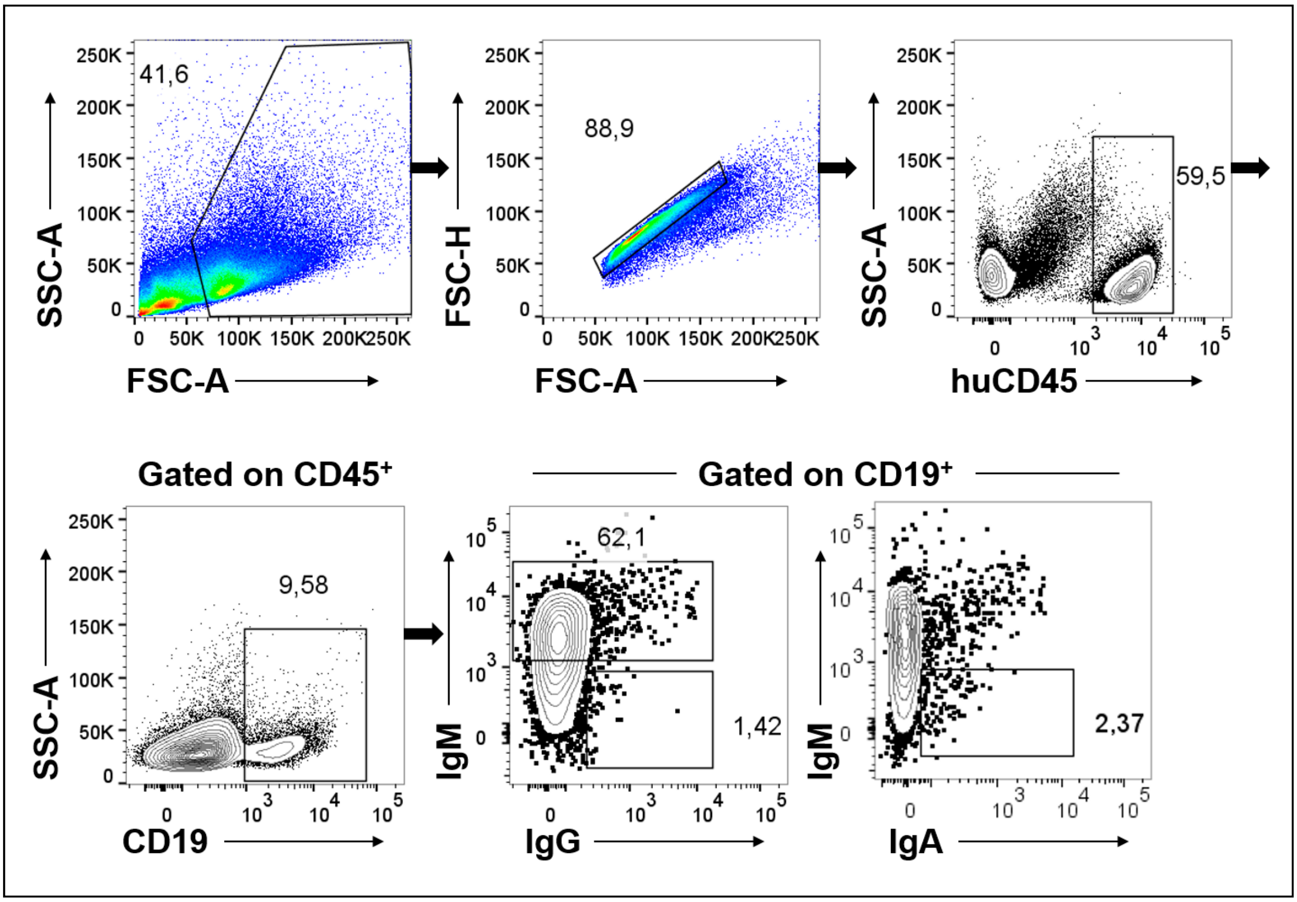

Figure S4. FACS gating strategy for analyses of B cell phenotype in spleen. Representative example of huNRG spleen analyses 20 weeks after HCT for an DR4/VAC mouse.
